# Functional and clinical characterization of the alternatively spliced isoform *AML1-ETO9a* in adult patients with translocation t(8;21)(q22;q22.1) acute myeloid leukemia (AML)

**DOI:** 10.1038/s41375-019-0551-4

**Published:** 2019-08-28

**Authors:** Mridul Agrawal, Peggy Schwarz, Benedetto Daniele Giaimo, Ivan Bedzhov, Andrea Corbacioglu, Daniela Weber, Verena I. Gaidzik, Nikolaus Jahn, Frank G. Rücker, Thomas Schroeder, Thomas Kindler, Mohammed Wattad, Katharina Götze, Michael Lübbert, Hans Salwender, Mark Ringhoffer, Elisabeth Lange, Elisabeth Koller, Felicitas Thol, Michael Heuser, Arnold Ganser, Lars Bullinger, Peter Paschka, Hartmut Döhner, Hartmut Geiger, Tilman Borggrefe, Konstanze Döhner, Franz Oswald

**Affiliations:** 1 Klinik für Innere Medizin III, Universitätsklinikum Ulm, Ulm, Germany; 2 Klinik für Innere Medizin I, Universitätsklinikum Ulm, Ulm, Germany; 3 Institute of Biochemistry, University of Giessen, Giessen, Germany; 4 Embryonic Self-Organization research group, Max Planck Institute for Molecular Biomedicine, Röntgenstraße 20 48149 Münster, Germany; 5 Klinik für Hämatologie, Onkologie und Klinische Immunologie, Universitätsklinikum Düsseldorf, Düsseldorf, Germany; 6 III. Medizinische Klinik und Poliklinik, Universitätsmedizin Mainz, Mainz, Germany; 7 Klinik für Hämatologie, Internistische Onkologie und Stammzellentransplantation, Evangelisches Krankenhaus Essen-Werden, Essen, Germany; 8 III. Medizinische Klinik, Klinikum rechts der Isar der Technischen Universität München, München, Germany; 9 Klinik für Innere Medizin I, Universitätsklinikum Freiburg, Freiburg, Germany; 10 II. Medizinische Abteilung, Asklepios Klinik Altona, Hamburg, Germany; 11 Medizinische Klinik III, Städtisches Klinikum Karlsruhe, Karlsruhe, Germany; 12 Klinik für Hämatologie, Onkologie und Palliativmedizin, Evangelisches Krankenhaus Hamm, Hamm, Germany; 13 Medizinische Abteilung, Hanusch-Krankenhaus der WGKK, Wien, Austria; 14 Klinik für Hämatologie, Hämostaseologie, Onkologie und Stammzelltransplantation, Medizinische Hochschule Hannover, Hannover, Germany; 15 Medizinische Klinik m. S. Hämatologie, Onkologie und Tumorimmunologie, Campus Virchow-Klinikum, Charité Universitätsmedizin Berlin, Berlin, Germany; 16 Institut für Molekulare Medizin, Universität Ulm, Ulm, Germany; 17 Division of Experimental Hematology and Cancer Biology, CCHMC, Cincinnati, OH USA

**Keywords:** Oncogenesis, Oncogenes

## To the Editor:

Acute myeloid leukemia (AML) encompassing translocation t(8;21)(q22;q22.1) results in the chimeric fusion protein AML1-ETO (AE), also known as *RUNX1-RUNX1T1* transcript. The presence of AE defines a precursor stage of leukemia, however additional molecular events are required for transformation [1, 2]. Alternative splicing of the *ETO* gene introduces an additional exon adjacent to exon 8, namely exon 9a, spanning 155 bp. Inclusion of exon 9a alters the open reading frame of the AE gene leading to a carboxy-terminal truncated isoform of the AE protein, known as AML1-ETO9a (AE9a), which lacks *Drosophila* nervy homology regions (NHR) 3 and 4 [3]. In a retroviral transduced mouse model, co-expression of AE and AE9a induces a more immature leukemic phenotype with a rapid onset of AML [4]. The authors hypothesized that the relative *AE9a* allelic burden as compared to the full-length *AE* transcript might affect the transforming capacity of the protein [4]. As to now, there is only scarce data on the incidence of AE9a with limited evidence indicating that *AE9a* transcript levels (TL) impact on prognosis in t(8;21)-AML. In a previous study on 118 pediatric and adult t(8;21)-AML patients, elevated *AE9a* (*n* = 86) was correlated with the worsened clinical outcome as well as increased incidence of *KIT* mutations and higher *KIT* gene expression [5]. However, the two-step nested PCR approach used to detect *AE9a* does not allow accurate quantification and therefore possibly overestimates gene expression levels. In another study, Ommen et al. reported the presence of *AE9a* in 11/13 patients with t(8;21)-AML and observed lower decline of *AE9a* TL in relapsing as compared to non-relapsing patients during the course of the disease [6]. More recently, we performed a transcriptome study applying novel high-throughput sequencing technologies and detected the *AE9a* variant in 27/27 t(8;21)-AML cases [7].

We, therefore, sought to systematically assess the incidence and prognostic significance of *AE9a* co-expression in the context of clinical and genetic factors in a large clinically well-annotated cohort of patients with t(8;21)-AML. We complemented these analyses by the generation of a mouse model (*Rosa26-LSL-AE9a-IRES-GFP x Vav1-Cre*) with hematopoietic-specific AE9a-expression starting early on in development to determine the role of AE9a for leukemia initiation and progression.

In total, 129 patients based on the availability of a diagnostic bone marrow (BM) or peripheral blood (PB) sample were included; 93 patients were enrolled on one of five clinical trial protocols of the German-Austrian AML Study Group (AMLSG) (Supplemental Appendix); 36 patients were treated outside clinical studies. 127 patients received standard intensive chemotherapy and 2 patients were treated based on a non-intensive treatment protocol. The median follow up was 3.6 years (detailed patients characteristics are provided in the Supplemental Appendix, Table S1). *AE9a* mRNA expression was determined by qRT-PCR (Fig. S1a). Co-expression of *AE9a* as a fraction of the full-length *AE* transcript was reported as *AE9a*/*AE* ratio (%); *ABL1* was used as housekeeping gene control [8]. Gene mutation status was available for *KIT*, *FLT3* (ITD/TKD), *NRA*S and *ASXL2* [9–11]. This study was conducted in accordance with the Declaration of Helsinki. Written informed consent for treatment and genetic testing was obtained from all patients.

The *AE9a* isoform was detectable in all 129 patients of our study cohort, which is in line with our recent finding and the data published by Ommen et al. [6, 7]. In contrast, Jiao et *al*. identified the *AE9a* splice variant only in a proportion of the patients [5]. This discrepancy is probably due to the varying techniques that have been applied; Jiao et al. determined the relative gene expression < 10^−3^ on a gradient dilution of Kasumi-1 cells as threshold for PCR-negativity [5], whereas our definition of PCR-negativity was set C_t_ > Y-intercept. In our data set, the median *AE9a*/*AE* ratio was 32% (range 3–77%) and did not significantly differ between BM (*n* = 116, range 8–77%, median 31%) and PB (*n* = 13, range 3–66%, median 52%). Interestingly, the allelic *AE9a* burden corresponded to our previous findings independently obtained by RNA-sequencing (13–64%) [7]. Median *AE9a*/*AE* ratio was neither correlated with clinical features (sex, age, WBC, platelets, BM blasts; Table S2) nor gene mutations affecting *KIT*, *FLT3* or *ASXL2*.

**Fig. 1:**
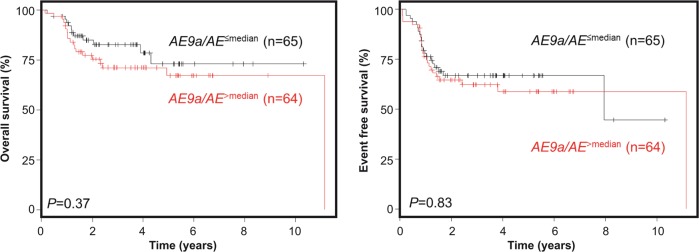
Prognostic impact of *AE9a/AE* on clinical outcome. OS (left) and EFS (right) are shown according to dichotomization of *AE9a/AE*^>median^ (red) and *AE9a/AE*^≤median^ (black)

Using Cox regression analysis, *AE9a*/*AE* ratios were not associated with the clinical endpoints overall survival (OS), event-free survival (EFS) and cumulative incidence of relapse (CIR) (Table S3). The same was true when we performed univariate analyses comparing *AE9a*/*AE* ratios dichotomized at the median (*AE9a*/*AE*^>median^ vs. *AE9a*/*AE*^≤median^): here, *AE9a*/*AE* ratios did not impact 4-yr OS (71 vs 79%; *P* = 0.37; Fig. 1 left, Table S4), 4-yr EFS (67 vs 59%; *P* = 0.83; Fig. 1 right, Table S4), and 4-yr CIR (32 vs 33%; *P* = 0.35; Table S4). Furthermore, we evaluated a possible correlation between *AE9a*/*AE* ratio and *NRAS* or *KIT* mutations that frequently co-occur in the t(8;21)-AML subtype. In a subgroup analysis, no significant differences with regard to the clinical endpoints OS, EFS and CIR were found between *AE9a*/*AE*^>median^ and *AE9a*/*AE*^≤median^ if stratified according to *NRAS* (Fig. S1b) or *KIT* (Fig. S1c) mutations. Finally, absolute *AE9a* quantification as ratio to the *ABL* housekeeping gene was performed to test its prognostic value independently of the *AE* transcript. *AE9a*/*ABL* ratios were dichotomized along the median (*AE9a*/*ABL*^>median^ vs. *AE9a*/*ABL*^≤median^) but again were not associated with outcome: 4-yr OS (74 vs 89%; *P* = 0.33), 4-yr EFS (58 vs 72%; *P* = 0.18), and 4-yr CIR (40 vs 29%; *P* = 0.31).

**Fig. 2:**
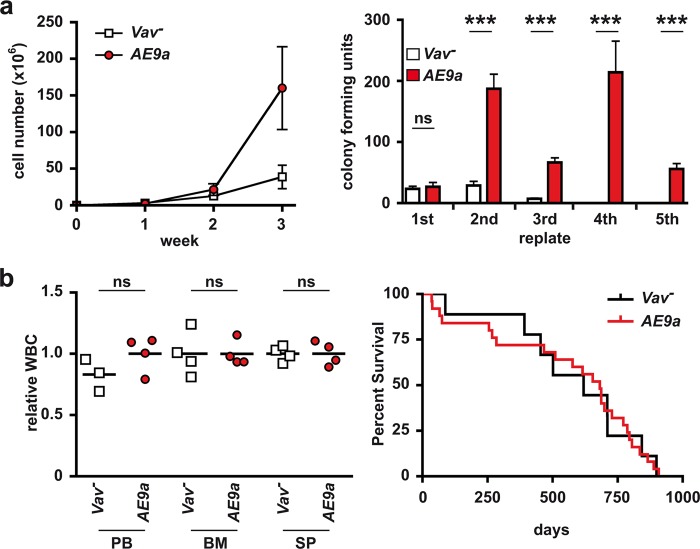
*AE9a*-expressing bone marrow cells exhibit enhanced stem cell characteristics but do not initiate leukemogenesis. **a**, left, *AE9a*-expressing cells show enhanced proliferation capacity. Proliferation potential of Lineage^−^, cKit^+^, GFP^+^ cells from 12 weeks old *AE9a* mice (red dots) and Lineage^−^, cKit^+^ cells isolated from Cre-negative littermate controls (*Vav*^−^, white squares) in suspension culture was estimated by cell number counts taken in seven days intervals over 3 weeks. MW  ± SD, *n* = 2. **a**, right, AE9a-expressing cells show significant self-renewal capacity. Colony-forming potential of Lineage^−^, cKit^+^, GFP^+^ cells from 12 weeks old *AE9a* mice (red bars) and Lineage^−^, cKit^+^ cells isolated from *Vav1*^−^ littermate controls (white bars) was measured by serial replating on semi-solid methylcellulose medium in seven days intervals over 5 weeks. MW ± SD (error bars) of the colony-forming units of triplicates from one representative experiment with *n* = 2 mice/group is shown. **b**, left, White blood cell counts (WBC) are not altered in 16 weeks old *AE9a* mice (red dots) compared to *Vav*^*−*^ littermate controls (white squares). Individual and mean values of peripheral blood (PB), bone marrow (BM) and spleen (SP) from *n* = 4 mice/group are shown relative to the mean of the respective *Vav*^−^ group. (**b**, right) *AE9a* expression in the hematopoietic compartment does not influence survival of mice. Kaplan–Meier plot illustrating that survival in *AE9a* mice (red line, mean survival 580 days, *n* = 25) is not altered compared to *Vav*^*−*^ control mice (black line, mean survival 559 days, *n* = 9). ns, not significant; ***p < 0.001

Recently, several studies showed that elevated expression of AE9a in primitive hematopoietic cells in mice via retroviral transduction protocols and subsequent transplantation of the transduced cells results in leukemia, even though with a long latency [12, 13]. These data imply a role for AE9a in leukemia initiation or progression, but do not exclude that additional mutations upon virus insertion might be necessary to contribute to the disease. In order to validate our clinical observations, we generated a novel mouse model for targeted expression of AE9a in the hematopoietic system (*Rosa26-LSL-AE9a-IRES-GFP* x *Vav1-Cre*, hereafter referred to as *AE9a* mice, Fig. S2a–c, Tables S5–S7) to further characterize the role of AE9a in leukemogenesis independent of viral transduction protocols. Expression of *AE9a* was verified at the level of mRNA (Fig. S2d), protein (Fig. S2e) as well as by GFP co-expression (Fig. S2f, g). As expected, isolated GFP^+^ BM cells from twelve weeks old *AE9a* mice (Fig. S3a) showed increased proliferation rates (Fig. 2a, left) and colony-forming capacity (Fig. 2a, right) compared to cells from control (*Vav*^-^) mice. In addition, numbers of short term hematopoietic stem cells (ST-HSCs, Fig. S3b, left) were significantly elevated, while there were no significant changes in the number of long term hematopoietic stem cells (LT-HSCs, Fig. S3b, middle) and LSK cells (Fig. S3b, right). In addition, the frequency of CMPs (common myeloid progenitors; CD16/32^−^, CD34^+^) and GMPs (granulocyte/macrophage progenitors, CD16/32^+^, CD34^+^), but not MEPs (megakaryocyte-erythrocyte progenitors, CD16/32^−^, CD34^−^) was elevated (Fig. S3c). Most interestingly, although there was a tendency for an elevated number of c-Kit^+^ cells in BM and spleen which usually correlates with pre-leukemia, [Fig. S3d [12, 13]], none of the AE9a-expressing mice showed signs of leukemia as revealed by WBC (Fig. 2b, left) or survival compared to the controls (Fig. 2b, right).

We here report on the occurrence and prognostic impact of the *AE9a* splice variant in the so far largest set of adult t(8;21)-AML patients. Using a sensitive and robust quantitative RT-PCR assay, *AE9a* was detectable in all patients. In contrast to the previous studies, our study was performed in a large cohort of uniformly treated patients. Neither *AE9a/AE*, nor *AE9a/ABL* ratios correlate with any clinical feature and they do not impact on clinical outcome. These clinical observations are in line with data generated by our novel murine model which unequivocally demonstrates that expression of AE9a might contribute to leukemogenesis but is clearly not sufficient for the initiation of leukemia in mice.

We have recently investigated the molecular mechanisms of AE9a-dependent transformation in a viral transduction/transplantation model by analyzing its dual role in deregulation of the AML1 activating and the ETO repressing gene regulatory functions. In that system, the deregulation of both *Notch* and *Aml1* target genes were required for the development of AE9a-driven leukemia [12] further supporting a necessary, but not sufficient role of AE9a for leukemia initiation. Thus, it is likely that a viral integration vector system for introducing AE9a in mice may cause leukemia through the activation of adjacent proto-oncogenes and therefore might not adequately recapitulate the human leukemogenesis.

In summary, in our large cohort of adult patients with t(8;21)-AML alternative splicing of the *AML1-ETO* fusion transcript represents a common feature. We could demonstrate that the allelic *AE9a* burden does not impact prognosis of this AML subtype therefore precluding its potential as a novel independent prognostic marker. Our clinical observation data were complemented by our recently established conditional *AE9a* knock-in mouse model showing that AE9a expression leads to enhanced proliferation and replating capacity but not to overt leukemia. Thus, AE9a rather acts as a precondition which requires a “second hit” for the development of AML. Alternative model systems like our tissue-specific knock-in mouse model may help to identify the critical “second hit” or additional environmental factors such as irradiation or chemotherapeutic agents.

## Supplementary information

Supplemental Appendix

Supplementary Figures
