# Supplementary material for: Functional and clinical characterization of the alternatively spliced isoform AML1-ETO9a in adult patients with translocation t(8;21)(q22;q22.1) acute myeloid leukemia (AML)
Source: Leukemia. 2019 Aug 28;34(2):630–4. doi: 10.1038/s41375-019-0551-4 (PMC7214266; doi:10.1038/s41375-019-0551-4)
Supplement: Supplementary file 1 — Supplemental Appendix [file 41375_2019_551_MOESM1_ESM.pdf]

Functional and Clinical Characterization of the Alternatively Spliced Isoform *AML1-ETO9a* in Adult Patients with translocation t(8;21)(q22;q22.1) Acute Myeloid Leukemia (AML)

Mridul Agrawal[1#], Peggy Schwarz[2#], Benedetto Daniele Giaimo[3], Ivan Bedzhov[4], Andrea Corbacioglu[1], Daniela Weber[1], Verena I. Gaidzik[1], Nikolaus Jahn[1], Frank G. Rücker[1], Thomas Schroeder[5], Thomas Kindler[6], Mohammed Wattad[7], Katharina Götze[8], Michael Lübbert[9], Hans Salwender[10], Mark Ringhoffer[11], Elisabeth Lange[12], Elisabeth Koller[13], Felicitas Thol[14], Michael Heuser[14], Arnold Ganser[14], Lars Bullinger[15], Peter Paschka[1], Hartmut Döhner[1], Hartmut Geiger[16, 17], Tilman Borggrefe[3], Konstanze Döhner[1*] and Franz Oswald[2*]

[1]Klinik für Innere Medizin III, Universitätsklinikum Ulm, Ulm, Germany; [2]Klinik für Innere Medizin I, Universitätsklinikum Ulm, Ulm, Germany; [3]Institute of Biochemistry, University of Giessen, Giessen, Germany; [4]Embryonic Self-Organization research group Max Planck Institute for Molecular Biomedicine Röntgenstraße 20, 48149 Münster, Germany [5]Klinik für Hämatologie, Onkologie und Klinische Immunologie, Universitätsklinikum Düsseldorf, Düsseldorf, Germany; [6]III. Medizinische Klinik und Poliklinik, Universitätsmedizin Mainz, Mainz, Germany; [7]Klinik für Hämatologie, Internistische Onkologie und Stammzellentransplantation, Evangelisches Krankenhaus Essen-Werden, Essen, Germany; [8]III. Medizinische Klinik, Klinikum rechts der Isar der Technischen Universität München, München, Germany; [9]Klinik für Innere Medizin I, Universitätsklinikum Freiburg, Freiburg, Germany [10]II. Medizinische Abteilung, Asklepios Klinik Altona, Hamburg, Germany; [11]Medizinische Klinik III, Städtisches Klinikum Karlsruhe, Karlsruhe, Germany; [12]Klinik für Hämatologie, Onkologie und Palliativmedizin, Evangelisches Krankenhaus Hamm, Hamm, Germany; [13]Medizinische Abteilung, Hanusch-Krankenhaus der WGKK, Wien, Austria; [14]Klinik für Hämatologie, Hämostaseologie, Onkologie und Stammzelltransplantation, Medizinische Hochschule Hannover, Hannover, Germany; [15]Medizinische Klinik m. S. Hämatologie, Onkologie und Tumorimmunologie, Campus Virchow-Klinikum, Charité Universitätsmedizin Berlin, Berlin, Germany, [16]Institut für Molekulare Medizin, Universität Ulm, Ulm, Germany; [17]Division of Experimental Hematology and Cancer Biology, CCHMC, Cincinnati, USA.

Running title: *AML1-ETO9a* in t(8;21)-AML.

Correspondence: Konstanze Döhner, MD, Department of Internal Medicine III, University Hospital of Ulm, Albert-Einstein-Allee 23, D-89081 Ulm, Germany. Phone: (+49) 731-500 45501, Fax: (+49) 731-500 45505, E-mail: konstanze.doehner@uniklinik-ulm.de

[#] MA and PS contributed equally to this study

*KD and FO share senior authorship

SUPPLEMENTAL APPENDIX

METHODS

Quantification of ***AML1-ETO9a***

qRT-PCR reactions were carried out using the MicroAmp™ Fast Optical 96-Well Reaction Plate (Applied Biosystems, Foster City, CA). The total volume was 25 µl based on the following protocol: 2.5 µl cDNA, 12.5 µl TaqMan® Universal PCR Master Mix (2 x), 3 pmol/µl of forward and reverse primers, as well as 3 pmol/µl of a FAM/BHQ2 labeled probe, respectively. In the case of *AE*, 1 pmol/µl of a FAM/TAMRA labeled probe was used. PCR conditions were: 50°C for 2 minutes and 95°C for 10 minutes, followed by 50 cycles at 95°C for 15 seconds and 60°C for 60 seconds. Primer, probe and qRT-PCR design of *AE9a* are provided in Table S5 and Figure S1. Primer and probes for the detection of *AE* were chosen based on the "A Europe Against Cancer Program" (EAC) protocol (1).

qRT-PCR analyses for *AE9a* and *AE* were performed in triplicate and included patient samples, negative template controls (NTC) and serial plasmid dilutions (10^2-10^6) for the respective genes. *ABL1* was quantified in duplicate, serial plasmid dilutions ranged from 10^2 to 10^4. QuantStudio 12K Flex Software (Applied Biosystems, Foster City, CA) was used for visualization of the amplification curves and C_t value calculation. The threshold for C_t values was set to 0.1 and baseline intervals were 3 to 15. The mean slope was -3.52 and the mean Y-intercept was 45.7 C_t. Negativity in all NTC and a correlation coefficient of the standard curve ≥0.99 was mandatory for further evaluation. qRT-PCR negativity was defined as C_t >Y-intercept.

Patients

93 of the 129 patients with t(8;21)-AML were enrolled on one of five clinical trial protocols of the German-Austrian AML Study Group (AMLSG): AML HD98A (NCT00146120; n=10), AMLSG 06-04 (NCT00151255; n=4), AMLSG 07-04 (NCT00151242; n=37), AMLSG 11-08 (NCT00850382; n=27) and AMLSG 21-13 (NCT02013648; n=15); 36 patients were treated outside clinical studies.

Statistical Analyses

The following statistical approaches were used for clinical outcome analyses: cumulative incidence of relapse (CIR) was calculated according to Kalbfleisch and Prentice, differences between two groups were analyzed using Gray's test (2) and the median follow-up for survival was determined according to the method of Korn (3). The definition of CR, event-free survival (EFS), relapse-free survival (RFS), and overall survival (OS) were based on consensus recommendations of the European LeukemiaNet (4). Correlations between continuous variables were calculated using the Spearman rank test. Kaplan-Meier analysis was applied for survival estimation, differences between two groups were reported using the two-sided log-rank test. An effect was considered significant in case the *P*-value was below 5%. All statistical analyses were performed with the statistical software environment R version 3.2.1, using the R packages Hmisc version 3.13-0, survival version 2.37-7 and cmprsk version 2.2-6.

Cell culture

Feeder cells and Embryonic Stem Cells (ESCs) were grown at 37°C with 5% CO_2 in DMEM (Biochrom, FG0435) supplemented with 15% FCS, 1% Pen/Strep (Gibco, 15070-63), 1 mM Na-Pyruvate (Gibco, 11360.039), 2 mM L-Glutamin (Gibco, 25030-024), 1x MEM-NEAA (Gibco, 1140-035) and β-mercaptoethanol (Sigma-Aldrich, M7522-100ML). Only in the case of the ESCs, the medium was supplemented with 500 U/ml LIF (ESGRO, ESG1107). To prevent the differentiation of the ESCs, MAPK inhibitor PD98059 and GSK3β inhibitor SB216763 were added to 50 μM and 5 μM, respectively (2I medium). Feeder cells were inactivated with 20 μg/ml of mitomycin C (Sigma-Aldrich MO503). ESCs were electroporated with the plasmid DNA of choice and seeded on inactive feeder cells at different dilutions. After 24 h incubation at 37°C, positively electroporated cells were selected with 500 μg/ml G418 (Sigma-Aldrich A1720-56). Single positive colonies were transferred to 96 wells plates in which inactive feeder cells were previously seeded. After replica of the 96 wells plates, one plate for each line was frozen at -80°C after adding DMSO and the other plates were expanded to purify the genomic DNA (gDNA) and perform screening via PCR and Southern Blotting.

Genomic DNA (gDNA) extraction, PCR and Southern Blotting

ESCs were washed with PBS and lysed in gDNA extraction buffer (10 mM Tris-HCl pH 7.5, 10 mM EDTA, 10 mM NaCl, 0.5% NLauroylsarcosine sodium salt, 1 mg/ml Proteinase K) overnight at 37°C. The gDNA was precipitated by adding NaCl/EtOH and washed with 70% EtOH. The pellet was finally resuspended in TE pH 8.0 and analyzed by PCR and Southern Blotting.

The gDNA was digested overnight using 50 U of the desired restriction enzyme (NEB) accordingly to manufacturer´s instructions and the digestion was subsequently extended for 6

more hours with 50 more U of the respective enzyme. The gDNA was resolved on a 0.6% agarose gel and subsequently incubated in Denaturation Buffer (1.5 M NaCl, 0.5 M NaOH) for 40 min at room temperature. After washing twice in Neutralization Buffer (1.5 M NaCl, 0.5 M Tris-HCl pH 7.0) for 20 min each at room temperature, the gDNA was transferred overnight to a Hybond-N membrane (Amersham, RPN203N) and cross-linked. To prepare the probes, 15 µg of the plasmids of choice were digested overnight with 20 U of the restriction enzyme (NEB) of choice (see Table S6) and purified from a 2% agarose gel with the Genelute gel extraction kit (Sigma-Aldrich) accordingly to manufacturer´s instructions. Probes were labeled with radioactive γ-32P dCTP (Hartman Analytic GmbH, SRP-205) using the Megaprime DNA labelling System (Amersham, RPN1606) accordingly to manufacturer's instructions and purified with Quick Spin Columns (Roche, Cat. No.11273965001) following manufacturer´s instructions.

For the hybridization of the membranes, the 5x MP-Mix [5% SDS (w/v), 5% milk (w/v)] was pre-heated at 56°C and the Pre-hybridization-Mix [4x SSC (20x stock solution, 3 M NaCl, 0.3 M trisodium citrate dihydrate pH 7.0), 1x MP-Mix, 300 µg/ml Salmon Sperm DNA (Sigma-Aldrich D1626)] was pre-heated at 100°C for 10 min. The membrane was blocked 4 hours at 65°C in Pre-hybridization-Mix. Labelled probes were added to the Hybridization-Mix [3.6x SSC, 0.9x MP-Mix, 450 µg/ml Salmon Sperm DNA, 18% Dextransulfate (w/v)] and denatured at 100°C for 10 minutes. The membrane was hybridized over night at 65°C and washed at 50°C in Washing Buffer [0.1x SSC, 0.05% SDS (w/v)]. Signals were detected with a light sensitive film.

For mouse genotyping purposes, ear patches or tail sections were digested overnight in 200 µl of Proteinase K solution (10 mM Tris-HCl pH 8.0, 5 mM EDTA, 1% SDS, 300 mM sodium acetate,

200 µg/ml Proteinase K). The day after, the gDNA was precipitated by adding 2-propanol and washed with 70% EtOH. Finally, the gDNA was resuspended in TE buffer and analyzed by PCR.

In the case of the screening of the ESCs, the Taq polymerase (Genaxxon, M3043-0.250) was used with primers "Rosa26 AE 9a fw" and "Rosa26 AE 9a rev" For mouse genotyping purposes the DreamTaq (Fermentas, EP0701) was used together with primers "oIMR8545" and "oIMR8546" to discriminate between homo- and heterozygotes or with primers "EGFP dir" and "TV rosa up" to discriminate between targeted and wild-type alleles. Primers are listed in Table S5.

Plasmids

The following plasmids were kindly provided by Drs. Hassan Jumaa and Martina Bach: pCR21-TOPO-EP-5’, TV-Rosa26-Fc(g)RI, TV-Rosa26-LMP1. Details on the targeting vector TV-Rosa26-Fc-AE9a-IRES-GFP are available on request. Briefly, an AE9a-IRES-GFP cassette was constructed as *AscI/AscI* fragment by PCR aided cloning. This AE9a-IRES-GFP fragment was then inserted into the TV-Rosa26-Fc(g)RI vector after *AscI* digestion. All Plasmids are listed in Table S6.

30 µg of each targeting plasmid were digested for at least 16 h with 50 U of AgeI (NEB) at 37°C and the digestion was subsequently extended for 2 more hours at 37°C with 10 more U of AgeI. The digestion was monitored on a 0.8% agarose gel and the linearized plasmids purified by phenol/chloroform extraction followed by precipitation with sodium acetate/EtOH. The linearized DNA was washed in 70% EtOH, air-dried, resuspended in PBS and used for electroporation of ESCs.

Mice

The successfully targeted ESCs were injected into C57BL/6 blastocysts by the Transgenic Mouse Unit of the Max Planck Institute of Immunobiology and Epigenetics in Freiburg im Breisgau (Germany). The injected blastocysts were transferred into recipient foster mothers and the agouti-colored chimera mice were mated to C57BL/6 mice to test the germ line transmission of the targeted locus (Table S7). To specifically express AE9a in the hematopoietic compartment, mice were crossed with *Vav1*-Cre mice (5). Mice were bred and housed in specific pathogen free conditions in accordance with institutional, state and federal guidelines on animal welfare. All animal experiments were carried out in cooperation with the animal facility at the University of Ulm in accordance with the Tierschutzgesetz §8, Abs. 1 und 3.

Isolation of bone marrow cells and splenocytes

Bone marrow cells were isolated from femora, tibiae and hips of the mice by grinding the bones with mortar and pestle in PBS (Life Technologies, 14190-094) containing 10% FCS (Sigma Aldrich, F2442,) and 1% Penicillin/Streptomycin (Pen/Strep, Gibco, 15140-122). Cells were subsequently washed with PBS containing 10% FCS and 1% Pen/Strep and filtered through a 40 µm cell strainer (Corning, 352340,) to remove remaining bone fragments.

Spleen was grinded through a 40 µm cell strainer into PBS containing 10% FCS and 1% Pen/Strep to obtain splenocytes that were subsequently washed and once more filtered through a 40 µm cell strainer.

Proliferation assay and colony formation assay

Mononuclear bone marrow cells were enriched by density gradient centrifugation (Histopaque, Sigma-Aldrich, 1083) and stained for cKit (anti-c-Kit-AlexaFluor700, clone ACK2, eBioscience) before fluorescence-activated cell scanner sorted on a FACSAria™ III (BD Biosciences, Heidelberg, Germany; Lineage-, cKit^{+} for *Vav*$^{-}$ mice or Lineage^{-}, cKit^{+}, GFP^{+} for *AE9a* mice). For the proliferation assay, each 30000 sorted cells/mouse were seeded in 6-well dishes and cultured in 5 ml DMEM medium supplemented with 15% FCS (Sigma Aldrich, F2442), 1 % Pen/Strep and recombinant mouse IL-3, IL-6 and SCF (final concentration 0.1 ng/ml, respectively, all ImmunoTools, Germany). After 7 days, cells were counted and 10% of them were re-seeded for additional 7days. This procedure was repeated twice. For the colony formation assay, triplicates of each 100 cells/mouse were seeded on methylcellulose-based semi-solid media (R&D Systems USA, HSC008) in 6-well dishes. Colony-forming progenitors were scored every 7 days and replated in new methylcellulose medium at a concentration of 1×10^{4} cells/well for a total of 5 weeks.

Flow cytometry

For the analyses of stem and progenitor cells, mononuclear bone marrow cells were isolated from femora, tibiae and hips of the mice after density gradient centrifugation (Histopaque, Sigma-Aldrich, 1083). After blocking with purified CD16/CD32 antibody (BioLegend, 101302), cells were incubated with a cocktail of biotinylated antibodies (Biotin anti-mouse Lineage Panel, 133307) directed against specific surface molecules to exclude Lin^{+} cells: anti-CD11b (clone M1/70), anti-B220 (clone RA3-6B2), anti-CD3 (clone 145-2C11) anti-Gr-1 (clone RB6-8C5), anti-Ter119 and Streptavidin-Brilliant Violet605 as secondary antibody (405229, all from BioLegend).

Subsequently, cells were incubated with anti-Sca-1-PE-Cy7 (clone D7, eBioscience), anti-c-Kit-AlexaFluor700 (clone ACK2, eBioscience), anti-CD127/IL7Rα-APC-eFluor780 (clone A7R34, eBioscience), anti CD16/CD32-eFluor450 (FcγR, clone 93, eBioscience), anti-Flt3-PE (clone A2F10, eBioscience), anti-CD34-eFluor660 (clone RAM34, eBioscience) as well as Streptavidin-Brilliant Violet605 (BioLegend) for 20 min on ice. Cells were washed with PBS to remove unbound antibodies and resuspended in propidium iodide solution (0.1 µl/ml in PBS, Sigma, P4864) to exclude dead cells in the subsequent analyses on a LSRFortessa™ II (BD Biosciences, Heidelberg, Germany). The following marker combinations were used to discriminate between HSC and progenitor cells: LT-HSC: Lin$^-$, Sca-1$^+$, c-Kit$^+$, Flt3$^-$, CD34$^-$; ST-HSC: Lin$^-$, Sca-1$^+$, c-Kit$^+$, Flt3$^-$, CD34$^+$; CMPs: Lin$^-$, Sca-1$^-$, c-Kit$^+$, FcgR^{low}, CD34$^+$; GMPs: Lin$^-$, Sca-1$^-$, c-Kit$^+$, FcgR$^+$, CD34$^+$; MEPs: Lin$^-$, Sca-1$^-$, c-Kit$^+$, FcgR$^-$, CD34$^-$. To assess the cKit$^+$ cell population in peripheral blood, bone marrow and spleen, cells were processed as stated above, blocked and incubated with anti-c-Kit-AlexaFluor700 (clone ACK2, eBioscience) for 20 min on ice prior to washing and resuspending in propidium iodide solution. Subsequently, cells were analyzed on a LSRFortessa™ II (BD Biosciences, Heidelberg, Germany).

Survival of Mice

The Kaplan–Meier survival curve and statistical analyses were performed using GraphPad Prism4 software (GraphPad Software, San Diego, CA, USA).

Western Blotting

For the generation of whole cell protein extracts from bone marrow, cells were pelleted and lysed in Laemmli buffer (0.35 M Tris HCl pH6.8, 10.28% SDS, 36% glycerine, 5% ß-Mercaptoethanol, 0.012% bromphenol blue) for 30 min on ice before remaining cell fragments were separated by centrifugation. After separation on 10 % SDS-polyacrylamide gels, the proteins were electrophoretically transferred to PVDF membranes (Millipore) for 1 h at 250 mA using a Tris-glycine buffer system. Membranes were blocked with 3% milk powder in PBS-T (0.1% Tween-20 in PBS) and incubated with the selected antibodies (α-AML1: Cell Signaling, 4334; α-GFP: Roche Diagnostics GmbH, 11814460001; α−β-Aktin:, Sigma Aldrich, A1978) accordingly to manufacturer's instructions.

Hemavet

White Blood cell counts (WBC) was taken from peripheral blood as well as bone marrow and splenic cell suspensions on a HEMAVET 950FS (Drew Scientific Inc, USA).

Quantitative RT-PCR of murine cells

Isolation of total cell RNA from mouse splenocytes was performed with the RNeasy Mini Kit (QIAGEN, Germany). Briefly, the splenic tissue was mechanically disrupted (Ultra-Thurrax, Janke & Kunkel, IKA Labortechnik, Germany) in RLT buffer (provided in the kit) and lysates were immediately transferred to QIAshredder columns to remove cellular debris. Subsequently, the RNA was isolated following the kit's instructions. For every probe, 1µg of RNA was transcribed to cDNA with the SuperScript^{TM} II Reverse Transcriptase Kit (Thermo Fisher Scientific Germany, 18064014) according to the manufacturer's protocol using random hexamer primers (Thermo

Fisher Scientific, 48190011). Specific mRNA expression levels of *AE9a* (primer sequence is given in Table S5) and the housekeeping gene β-Actin (QuantiTect Primer Assay QIAGEN, QT01136772,) were analyzed on a 7500 Fast Real-Time PCR System (Applied Biosystems, Germany) with the RT2 SYBR® Green qPCR Mastermix (QIAGEN, 330529) and calculated with the 7500 Fast System SDS Software (Applied Biosystems).

SUPPLEMENTAL TABLES

Table S1. Patient characteristics.

	**Patients with *RUNX1-RUNX1*-positive AML (n=129)**
**Median age, years (range)**	49 (18 – 75)
**Male sex, n (%)**	67 (52)
**AML history, n (%)**	
De novo	114 (90)
**Secondary**	2 (2)
**Therapy-related**	11 (9)
**Missing data, n**	2
**Median WBC, x 10^9/l (range)**	9.2 (1.2 – 117.5)
**Missing data, n**	4
**Median platelet count, x 10^9/l (range)**	28 (3 - 303)
**Missing data, n**	4
**Median hemoglobin, g/dl (range)**	8.7 (3.8 – 15.1)
**Missing data, n**	4
**Median peripheral blood blasts, % (range)**	41 (0 - 99)
**Missing data, n**	10
**Median bone marrow blasts, % (range)**	60 (6 - 100)
**Missing data, n**	7
**Median LDH, U/l (range)**	466 (5 - 10823)
**Missing data, n**	2
***KIT* mutation, n (%)**	38 (30)
**Missing data, n**	3
***FLT3*-ITD/TKD mutation, n (%)**	12 (9)
**Missing data, n**	0
***NRAS* mutation, n (%)**	16 (13)
**Missing data, n**	2
***ASXL2* mutation, n (%)**	20 (19)
**Missing data, n**	26
**Double induction treatment, n (%)**	82 (64)
**Single induction treatment, n (%)**	45 (35)
**Non-intensive induction treatment, n (%)**	2 (2)

**Consolidation treatment, n (%)**	124 (96)
**autoHSCT**	2 (2)
**alloHSCT***	7 (5)
**HiDAC**	115 (89)

Table S2. Correlation of median *AE9a/AE* ratio with clinical features.

	**Rho (Spearman)**	***P***
Age	0.04	0.68
WBC	0.07	0.44
Platelets	0.02	0.79
LDH	-0.18	0.045
BM blasts	-0.06	0.52

Abbreviations: WBC, white blood cell count; LDH, lactate dehydrogenase; BM, bone marrow; *P*, p-value.

Table S3. Impact of *AE9a/AE* ratios on survival endpoints (Cox regression analysis).

	**OS**		**EFS**		**CIR**	
	HR	*P*	HR	*P*	HR	*P*
***AE9a/AE* ratios**	0.99	0.99	1.21	0.82	0.92	0.93

Abbreviations: OS, overall survival; EFS, event-free survival; CIR, cumulative incidence of relapse; *P*, p-value. NOTE: Analyses stratified by study

Table S4. Prognostic impact of *AE9a/AE* ratios dichotomized at the median on clinical outcome.

	**OS (%)**		**EFS (%)**		**CIR (%)**	
		P		***P***		***P***
AE9a/AE $^{>median}$	71	0.37	67	0.83	32	0.35
AE9a/AE $^{\leq median}$	79		59		33	

Abbreviations: OS, overall survival; EFS, event-free survival; CIR, cumulative incidence of relapse; *P*, p-value.

Table S5. PCR primers and probes used in this study.

Oligonucleotide	Sequence
**AE9a mRNA quantification (patients)**	
**AML-ETO-CFP**	5’-TCC GGC GGT ACA GTG A-3’
**AML-ETO-89R**	5’-CCG ATG CGC GTC TAG T-3’
**AML-ETO-9AR**	5’-AAG CTG GGC AGC ACC TA-3’
**AML-ETO-CP**	5’-AGG CAG CAG AGT CCC GTC AAC CC-3’ 5’FAM, 3’-BHQ2
**ESCs screening**	
**Rosa26 AE 9a fw**	5’-CCT CAG AGA GCC TCG GCT A-3’
**Rosa26 AE 9a rev**	5’-GGA CAG GAT AAG TAT GAC ATC A-3’
**Mouse genotyping**	
**oIMR8545**	5’-AAA GTC GCT CTG AGT TGT TAT-3’
**oIMR8546**	5’-GGA GCG GGA GAA ATG GAT ATG-3’
**EGFP dir**	5’-GGT GGT GCC CAT CCT GGT CG-3’
**TV rosa up**	5’-CCA GAT GAC TAC CTA TCC TCC-3’
**AE9a mRNA quantification (mice)**	
**RT_AE9a_up**	5’-AGT CGC CAC CTA CCA CAG AG-3’
**RT_AE9a_do**	5’-CTT CAC ATC CAC AGG TGA GTC TG-3’

Abbreviations: AML-ETO-CFP, common forward primer; AML-ETO-89R, reverse primer spanning fragments of *ETO* exon 8 and 9; AML-ETO-9AR, reverse primer containing fragments of *ETO* exon 9a; AML-ETO-CP, common probe.

Table S6. Plasmids used for targeting and to generate the probes for Southern blotting. The location of the probes on the targeted *ROSA26* locus and the restriction enzymes used to generate the probes are also indicated.

**Probe plasmids**		
**Plasmid**	**Probe**	**Enzymes**
pCR21-TOPO-EP-5'	short arm probe (Probe C)	XhoI/SacI
TV-Rosa26-Fc(g)RI	internal probe (Probe B)	NcoI/MluI
TV-Rosa26-LMP1	long arm (Probe A)	HindIII
**Targeting plasmids**		
**Plasmid**		
TV-Rosa-Fc IRES GFP		
TV-Rosa-Fc AE9a IRES GFP	see text	AscI/AscI

Table S7. Results of the injections of ESCs targeted with AE9a and grown in 2I medium.

	**No. transferred embryos**	**No. pups born (% of embryos)**	**% of chimerism**	**No. germ line transmission**
**AE9a 1D**	120	7 (5.8%)	0, 0, 85, 95, 100, 100, 100	1
**AE9a 6B**	106	14 (13.2%)	100, 95, 75, 85, 100, 100, 95, 95, 75, 85, 85,85, 85, 95	0
**AE9a 6D**	63	9 (14.3%)	100, 90, 100, 85, 95, 100, 100, 100, 100	5
**AE9a 7C**	60	10 (16.7%)	95, 90, 100, 85, 100, 100, 75, 70, 85	3

No., numbers

SUPPLEMENTAL FIGURE LEGENDS

Figure S1. (a) Schematic overview of *AE9a* and PCR design. Abbreviations: F, forward primer; P, PCR probe; R9a, reverse primer containing exon 9a fragments; R9, reverse primer containing exon 8 and 9 fragments; bp, base pairs. (b and c) Prognostic impact of AE9a/AE ratio on clinical outcome according to signaling gene mutation status. (b) OS in patients with *NRAS* wild-type (*NRAS*^{WT}, left), and *NRAS* mutated (*NRAS*^{mut}, right). (c) OS in patients with *KIT* wild-type (*KIT*^{WT}, left) and *KIT* mutated (*KIT*^{mut}, right). OS is shown according to dichotomization of AE9a/AE$^{>median}$ (red) and AE9a/AE$^{\leq median}$ (black).

Figure S2. Generation and characterization of an inducible *AE9a* expressing mouse model. (a) Strategy used to target the *ROSA26* locus in ESCs. The wild-type (WT) locus is shown (upper panel) together with the targeting vector (middle panel) and the recombinant locus (lower panel). The restriction sites (*Pvu*II and *Bam*HI) used for the screen are shown together with the probes used for the Southern blotting screen and the primers (grey bars) used for the PCR screen. (b) PCR screen of the ESCs electroporated with an empty vector or with a vector expressing *AE9a*. Positive targeting is indicated by a product of approximately 1.2 kb (primers are shown as arrows in a, lower panel). (c) Southern blotting of the ESCs electroporated with an empty vector or with a vector expressing *AE9a*. The gDNA was digested with the indicated restriction enzymes and analyzed with the probes indicated in the figure and depicted in panel a (lower panel). (d-g) *AE9a* and *GFP* are exclusively expressed in *AE9a* mice. (d) Total RNA of splenocytes isolated from 10 weeks old *AE9a* mice (red bar) and *Vav*$^{-}$ control mice (white bar) was reverse transcribed into cDNA and analyzed via qPCR utilizing a primer pair bridging the

fusion point of *AE9a*. Data obtained were normalized to the house keeping gene *β-Actin* and are shown as mean and standard deviation of duplicate experiments. *** p<0.001. (e) Western Blotting of bone marrow cells from 10 weeks old *AE9a* mice and *Vav*$^{-}$ control mice utilizing antibodies against AML1 (upper panel), GFP (middle panel) and β-Actin (lower panel) specifically detected AE9a (upper arrow) as well as GFP in *AE9a* mice but not in *Vav*$^{-}$ control mice. (f and g) Flow cytometric analyses of the GFP expression as a surrogate for the AE9a expression. Exemplary histograms of bone marrow cells from 10 weeks old mice shown in (f) illustrate that GFP expression specifically distinguishes *AE9a* mice (right panel, 86% of viable cells are GFP^{+}) from *Vav*$^{-}$ littermate controls (left panel, 1% of viable cells are GFP^{+}). The table in (g) summarizes the means (± SD) and the ranges of the GFP^{+} population (expressed as `% of viable cells´ in peripheral blood (PB), bone marrow (BM) and spleen (SP) in *Vav*$^{-}$ control mice and *AE9a* mice). n>15 mice/group/tissue.

Figure S3. Characterization of the leukemogenic potential of the *AE9a* expression mouse model. (a) Schematic overview of the experimental setup for the proliferation assay and the colony formation assay shown in Figure 2. Low density bone marrow cells were isolated from 12 weeks old *AE9a* mice and *Vav*$^{-}$ littermate controls, FACS sorted for Lineage^{-}, cKit^{+} cells (*Vav*$^{-}$ controls) or Lineage^{-}, cKit^{+}, GFP^{+} cells (*AE9a* mice) and subsequently subjected to the above mentioned assays. (b) Flow cytometric measurement of the hematopoietic stem cell pools in 16 weeks old *AE9a* mice and *Vav*$^{-}$ controls. While LSK (Lineage^{-},Sca-1^{+},cKit^{+}, right panel) and long-term hematopoietic stem cell (LT-HSC; LSK, CD34^{-}, Flt3^{-}, middle panel) pools show no differences between the analyzed groups, the short-term hematopoietic stem cell (ST-HSC; LSK, CD34^{+}, Flt3^{-},

left panel) pool is increased in *AE9a* mice (red dots) compared to *Vav*$^{-}$ littermate controls (white squares). Individual and mean values (expressed as `% of viable cells´, respectively) from n=4 mice/group are shown. (c) Increased progenitor cell pools in *AE9a* mice. Flow cytometric analysis of the hematopoietic progenitor cell pools in the bone marrow of 16 weeks old *AE9a* mice (red dots) and *Vav*$^{-}$ littermate controls (white squares) revealed increased common myeloid progenitors (CMP; Lineage^{-}, Sca1^{-}, cKit^{+} [LS-K], CD34^{+}, FcG^{med}, left panel) and granulocyte/macrophage progenitors (GMP; LS-K, CD34^{+}, FcG^{+}, middle panel) but not megakaryocyte-erythrocyte progenitors (MEP; LS-K, CD34^{+}, FcG^{-}, right panel) cell pools in *AE9a* mice. Individual and mean values (expressed as `% of viable cells´, respectively) from n=4 mice/group are shown. (d) The cKit^{+} populations are not increased upon *AE9a* expression. Flow cytometric measurement of the cKit^{+} population in peripheral blood (PB), bone marrow (BM) and spleen (SP) are not altered in 16 weeks old *AE9a* mice (red dots) compared to *Vav*$^{-}$ littermate controls (white squares). Individual and mean values (expressed as `% of viable cells´ from n=4 mice/group) are shown. ns, not significant; * $p<0.05$.

SUPPLEMENTAL REFERENCES

1. Gabert J, Beillard E, van der Velden VH, Bi W, Grimwade D, Pallisgaard N, et al. Standardization and quality control studies of 'real-time' quantitative reverse transcriptase polymerase chain reaction of fusion gene transcripts for residual disease detection in leukemia - a Europe Against Cancer program. *Leukemia* 2003; **17**: 2318-57.
2. Gray R. A class of *K*-sample tests for comparing the cumulative incidence of a competing risk. *Ann Stat* 1988; **16**: 1141-54.
3. Korn EL. Censoring distributions as a measure of follow-up in survival analysis. *Stat Med* 1986; **5**: 255-60.
4. Döhner H, Estey EH, Amadori S, Appelbaum FT, Büchner T, Burnett AK, et al. Diagnosis and management of acute myeloid leukemia in adults: recommendations from an international expert panel, on behalf of the European LeukemiaNet. *Blood* 2010; **115**: 453-74.
5. Croker BA, Metcalf D, Robb L, Wei W, Mifsud S, DiRago L et al. SOCS3 is a critical physiological negative regulator of G-CSF signaling and emergency granulopoiesis. *Immunity* 2004; **20**:153-165.
