# Supplementary material for: Functional and clinical characterization of the alternatively spliced isoform AML1-ETO9a in adult patients with translocation t(8;21)(q22;q22.1) acute myeloid leukemia (AML)
Source: Leukemia. 2019 Aug 28;34(2):630–4. doi: 10.1038/s41375-019-0551-4 (PMC7214266; doi:10.1038/s41375-019-0551-4)
Supplement: Supplementary file 2 — Supplementary Figures [file 41375_2019_551_MOESM2_ESM.pdf]

Figure S1

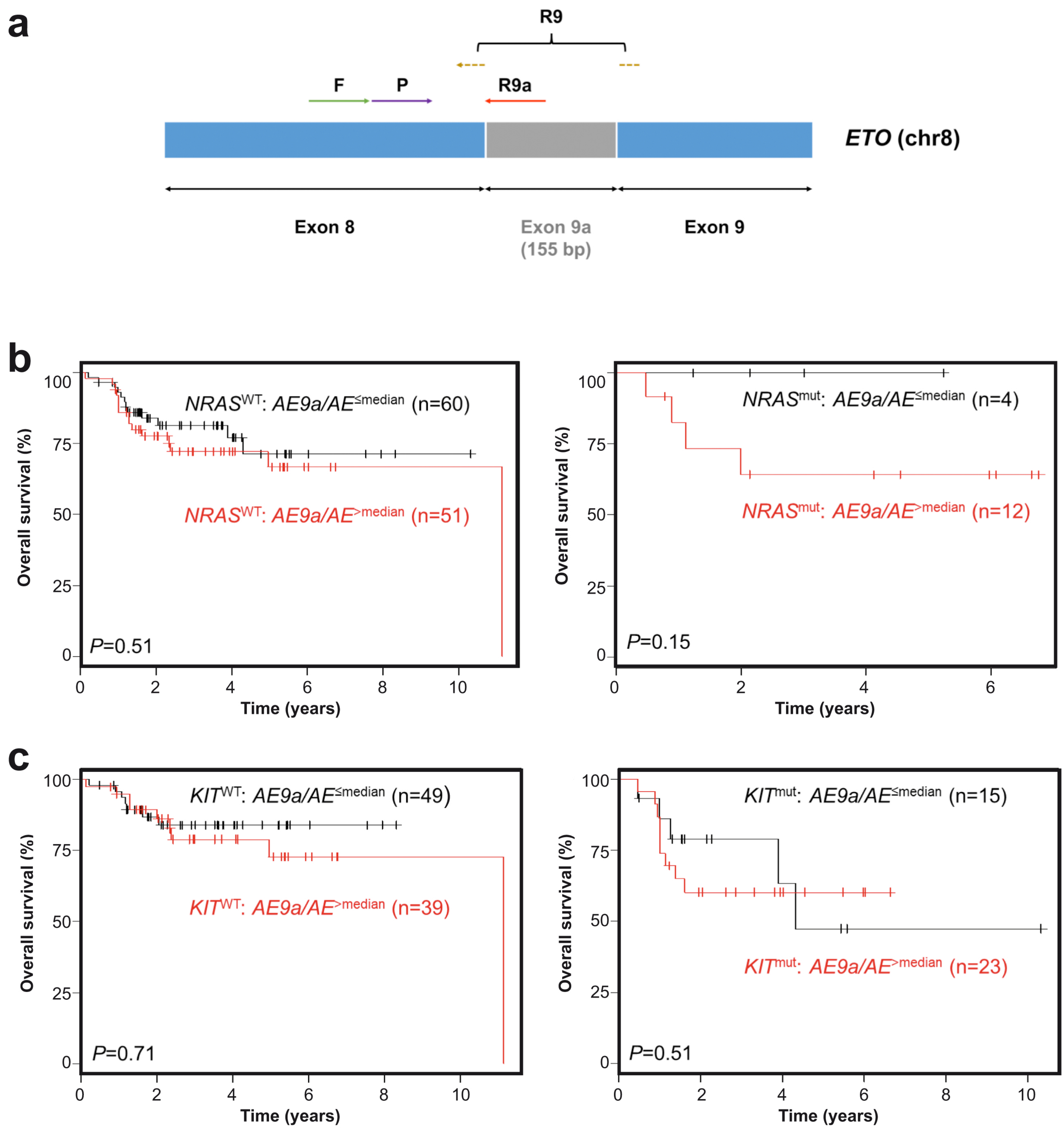
a
R9
F
P
R9a
ETO (chr8)
Exon 8
Exon 9a
(155 bp)
Exon 9
b
Overall survival (%)
Time (years)
NRASWT: AE9a/AE≤median (n=60)
NRASWT: AE9a/AE>median (n=51)
P=0.51
NRASmut: AE9a/AE≤median (n=4)
NRASmut: AE9a/AE>median (n=12)
P=0.15
c
KITWT: AE9a/AE≤median (n=49)
KITWT: AE9a/AE>median (n=39)
P=0.71
KITmut: AE9a/AE≤median (n=15)
KITmut: AE9a/AE>median (n=23)
P=0.51

Figure S2

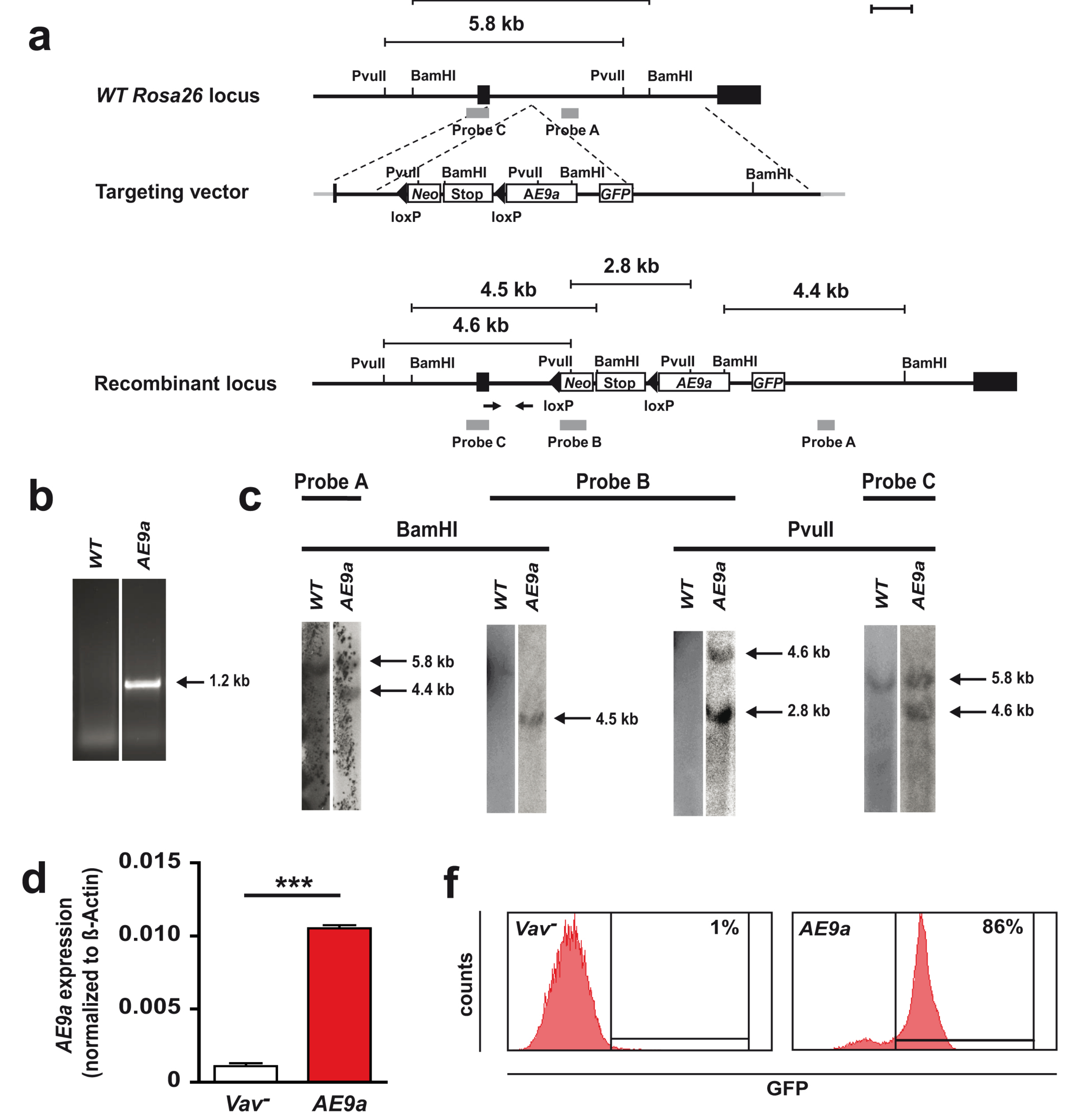

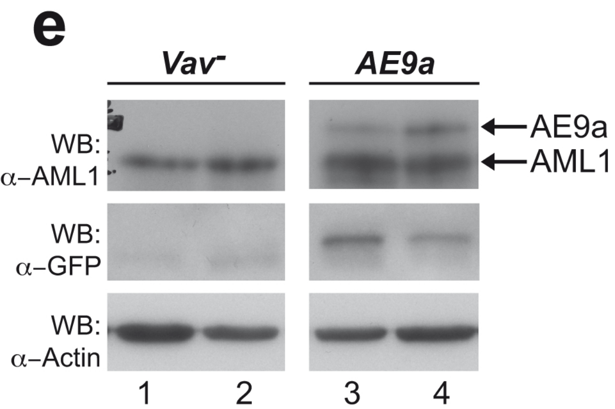

g

		PB	BM	SP
Vav⁻	mean (±SD)	1.0 (±1.0)	1.1 (±0.8)	2.1 (±1.5)
	range	0.1 - 3.2	0.2 - 2.9	0.4 - 5.4
AE9a	mean (±SD)	69.2 (±13.2)	88.7 (±6.5)	63.7 (±13.5)
	range	43.9 - 94.2	73.5 - 97.2	39.4 - 87.1
		p<0.001	p<0.001	p<0.001

Figure S3

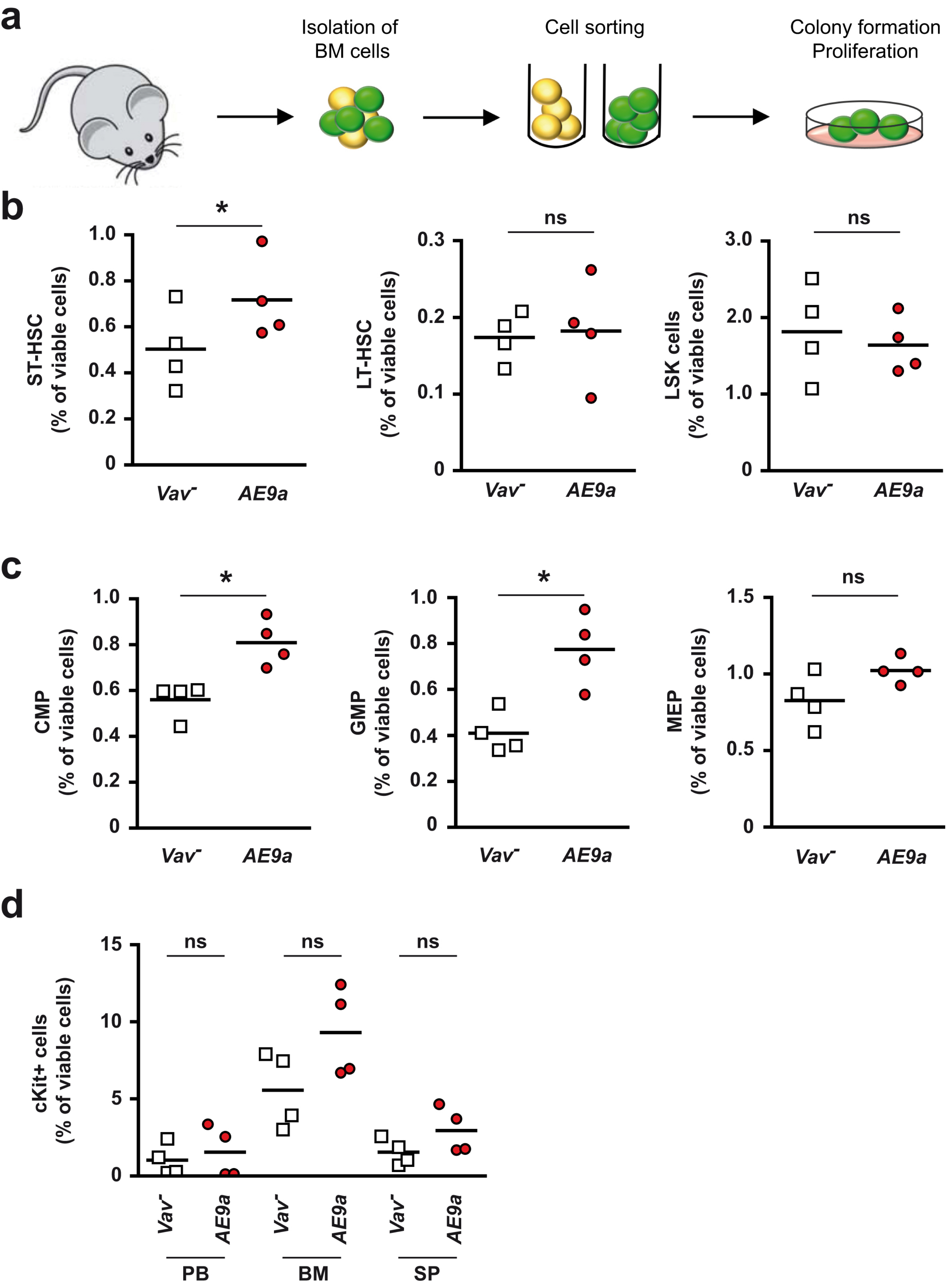

a
Isolation of BM cells
Cell sorting
Colony formation Proliferation
b
ST-HSC (% of viable cells)
LT-HSC (% of viable cells)
LSK cells (% of viable cells)
*
ns
ns
Vav⁻
AE9a
c
CMP (% of viable cells)
GMP (% of viable cells)
MEP (% of viable cells)
*
*
ns
d
cKit+ cells (% of viable cells)
ns
ns
ns
PB
BM
SP
